# Clinical and radiographic evaluation of premixed bioceramic putty as an apical plug in nonvital immature anterior permanent teeth

**DOI:** 10.1038/s41598-025-11407-x

**Published:** 2025-07-21

**Authors:** Mohamed S. Ghaly, Nura I. Abozena, Rehab F. Ghouraba, Ibrahim A. Kabbash, Shaimaa S. EL-Desouky

**Affiliations:** 1 Pediatric Dentistry, Oral Health and Preventive Dentistry Department, Faculty of Dentistry, Tanta University, Tanta, Egypt; 2 Oral Medicine, Periodontology, Oral Diagnosis, and Radiology Department, Faculty of Dentistry, Tanta University, Tanta, Egypt; 3 Public Health & Community Medicine Department, Faculty of Medicine, Tanta University, Tanta, Egypt

**Keywords:** Apexification, Apical plugs, Mineral trioxide aggregate, Well-Root PT, Biological techniques, Medical research, Materials science

## Abstract

Achieving an apical seal is critical for apexification treatment of nonvital immature teeth. While this is commonly accomplished using biocompatible mineral trioxide aggregate (MTA), its limitations, such as prolonged setting time, discoloration, and challenging handling, have driven the search for alternative materials. This study aimed to compare the clinical and radiographic success of bioceramic putty Well-Root PT apical plug compared to MTA in the treatment of nonvital immature permanent incisors. Fifty immature nonvital maxillary permanent central incisors in thirty-eight children aged 8–11 years were randomly divided into two groups (25 teeth/group). Group I received MTA apical plugs, and Group II was treated with Well-Root PT apical plugs. Both groups were recalled at 6 and 12 months for clinical and radiographic evaluations. Statistical analysis was done for the gathered data. Both groups showed improved clinical signs and symptoms during all follow-up periods with no statistically significant difference. Regarding the periapical radiolucency (PAR) area, at twelve months, the mean PAR area in the Well-Root PT group was (0.14 ± 0.08) compared to (2.3 ± 0.9) in the MTA group, with highly statistically significant differences (p < 0.001). The mean periapical bone radiodensity in the Well-Root PT group was (178.2 ± 5.4) compared to (164.8 ± 9.4) in the MTA group at twelve-month follow-up, with highly statistically significant differences(p < 0.001). Well-Root PT, with its reduced technical sensitivity, demonstrates satisfactory clinical and radiographic success as an apical plug for nonvital immature permanent incisors compared to MTA.

## Introduction

Young permanent teeth with incomplete root development and apical closure may lose their vitality due to traumatic dental injuries or dental decay^1^. The thin, brittle canal walls and diverging open apex of young permanent teeth with a necrotic pulp make traditional root canal treatment challenging to execute. Therefore, apexification and regenerative endodontics are typically the recommended course of action when the pulps of immature permanent teeth become necrotic^2,3^. Regenerative endodontics refers to biological techniques that replace damaged tooth structures, such as dentin and root structures, as well as pulp-dentin complex cells^3^. While apexification is defined as “a method to induce a calcified barrier in a root with an open apex or the continued apical development of an incomplete root in teeth with necrotic pulp”^1^, with success rates ranging from 81–100% after 1–15 years of follow-up^4^.

As stated by Kim et al.^5^, regenerative endodontic is indicated for short roots with thin canal walls, a wide-open apex, and teeth without the capacity for root formation, whereas apexification is done for teeth with nearly completed root formation and an open apex. Also, in a systematic review conducted by Panda et al.^6^, it found that both procedures (apexification and regenerative endodontics) have comparable survival rates; however, regenerative endodontics should be favored in cases where root growth is very poor, there is insufficient dentine, and the tooth’s prognosis is bleak even with an apexification procedure. Recently, Zanjir et al.^3^, created a core outcome set for endodontics (COS-ENDO) to measure outcomes in apexification and regenerative endodontic studies of permanent dentition and it was concluded that standardization of outcome reporting facilitates the integration of results across research syntheses, enhancing the understanding of the efficacy and effectiveness of various interventions influenced by procedural techniques, materials, and patient conditions.

Traditionally, calcium hydroxide was used in apexification by Kaiser and Frank in 1960^7^ to trigger the development of calcified tissues at the root apex. Despite its effectiveness and reliable outcomes, calcium hydroxide has several clinical limitations, including the long time required for calcified tissue formation, the need for multiple visits and consistent patient compliance, the risk of coronal leakage and reinfection from temporary restoration failure between appointments, and the tooth’s vulnerability to fractures^8^. The nature of the created barrier is permeable while being calcified, and occasionally even discovered to include tiny quantities of soft tissue^9^. These shortcomings have been addressed with the introduction of new alternative materials.

Looking for better choices has resulted in using mineral trioxide aggregate (MTA) as a highly promising material for apexification in one visit. MTA has excellent sealing properties and encourages peri-radicular tissue repair^10^. Nevertheless, MTA has a lot of drawbacks, such as difficult handling, a lengthy setting period, the possibility of discoloring teeth, and a high price. Regarding children’s teeth, the prolonged MTA setting time is a major disadvantage^11^.

The unique premixed bioceramics comprise “calcium silicates, zirconium oxide, tantalum oxide, calcium phosphate monobasic, and fillers” and possess superior handling characteristics. Additionally, they have good mechanical and biological qualities comparable to MTA biocompatibility for pulp tissue and can stimulate dentin bridge development^12,13^. Premixed bioceramics are hydrophilic, resistant to moisture and blood contamination, and are technique-insensitive^14^. Its hydration mechanism, which initially generates calcium hydroxide and dissociates into calcium and hydroxyl ions, causes the pH to be higher than 12 once set^15^. Well-Root PT is one of the novel premixed bioceramic materials supplied in the dental market^16^. This material possesses physicochemical, biological, and mechanical qualities similar to MTA and Biodentine^17^. Since Well-Root PT is accessible in premixed capsule form, it has the potential to provide uniform and appropriate consistency as well as clinical simplicity; it can be injected directly into the oral cavity by placing the capsule in a gun^18^. Also, it has a shorter setting time than MTA, with an initial setting time of about 5 min and a final setting time of 45 min^19^. Also, in a study conducted by Jeon et al.^20^, for color stability of pulp-capping materials, it was concluded that over time, Proroot White MTA and TheraCal LC demonstrated a notable drop in the L* value and an increase in the ΔE* value; conversely, Biodentine and Well-Root PT maintained a constant ΔE* value and showed no discernible change in the L* value, indicating no significant color change over time.

Further studies are necessary to evaluate Well-Root PT as an apical plug in nonvital immature permanent teeth. Therefore, this study aimed to compare the clinical and radiographic success of Well-Root PT apical plug compared to MTA in the treatment of nonvital immature permanent incisors. The null hypothesis (H0) proposed that there was no significant difference between Well-Root PT bioceramic putty and MTA when used as apical plugs in nonvital immature permanent incisors in terms of clinical success, periapical healing, and bone radiodensity over the follow-up period.

## Materials and methods

### Study setting and ethical considerations

A prospective double-blinded, controlled randomized clinical trial was conducted at the Outpatient Clinics of Paediatric Dentistry Department, Faculty of Dentistry, Tanta University, from November 2023 to December 2024. The digital X-ray was obtained at Tanta University’s Faculty of Dentistry, Oral Medicine and Periodontology, Oral Diagnosis & Oral Radiology Department. This trial was authorized by the ethical committee (REC), Faculty of Dentistry, Tanta University, code: # R-PED-11-23-3074, in accordance with the 1964 Helsinki Declaration and its following revisions, and was registered at ClinicalTrials.gov identifier NCT06322979 on (21/3/2024). The clinical treatment commenced after obtaining written informed consent from all participants and their legal guardians.

### Eligibility criteria

**Fig. 1:**
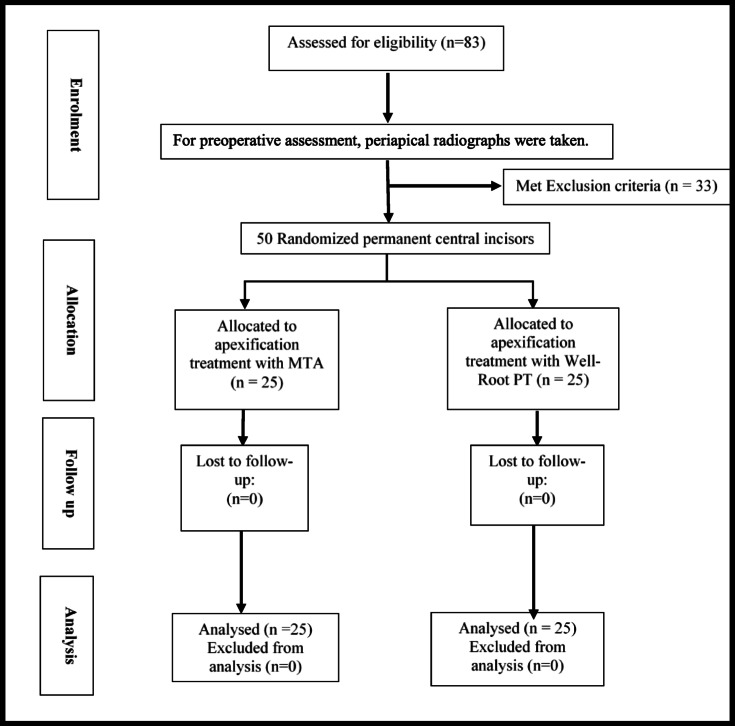
Flowchart detailing the assignment and randomization of the pediatric patients during the study.

Eighty-three nonvital maxillary permanent central incisors that resulted from dental caries or trauma in sixty children aged 8–11 years were recruited and assessed utilizing the inclusion and exclusion criteria of the study. Seemingly healthy cooperative children having nonvital clinically restorable one or both maxillary permanent central incisors with immature root end development as well as pulp necrosis, radiographic evidence of chronic apical periodontitis, and periapical radiolucency were included. Uncooperative patients or those with systemic disorders that affected their overall immunological condition were not allowed to participate in the study. Additionally, non-restorable teeth with significant internal/external pathological root resorption and/or extreme mobility were not included^21^. Therefore, thirty-three maxillary permanent central incisors were excluded, so the final study sample included fifty incisors in thirty-eight children. Figure 1 shows a flow chart comprising enrolment, allocation, assessment, and sample size analysis. Before treatment, all clinical and radiographic signs and symptoms were documented during the patient evaluation then all selected teeth underwent pulp vitality testing.

### Sample size calculation and randomization

The Epi-Info statistical program (version 7.2.6, 2023), developed by the World Health Organisation and Centres for Disease Control and Prevention, Atlanta, Georgia, USA, was used to determine the sample size and power analysis. A 95% confidence limit, 80% study power, and a predicted success rate of 60% for apical plugs in non-vital immature anterior permanent teeth in the least treatment group versus 95% in the most favorable treatment groups were the parameters utilized to calculate the sample size. The sample size for each group determined by the aforementioned criteria, was *N* = 23; it was increased to 25 permanent central incisors to compensate for insufficient outcomes and default rate.

Research Randomizer software program (https://www.randomizer.org/)^22^ was used for the randomization process. An autonomous individual generated a computer-randomized list, which was securely stored in a sealed, opaque envelope, to allocate participants meeting the inclusion criteria into one of the two groups.

### Group assignment

Fifty immature non-vital permanent maxillary central incisors were allotted randomly into two groups (25 tooth/group) based on treatment materials:

- **Group-I (control group)** (*n* = 25): 25 immature non-vital permanent central incisors with open apex received apexification treatment with MTA (MTA^+^, Cerkamed, Poland).
- **Group-II (experimental group)** (*n* = 25): 25 immature non-vital permanent central incisors with open apex received apexification treatment with Premixed Bioceramic Putty (Well-Root PT, Vericom Co., Korea).

### Apexification procedure

An apexification technique was performed under rigorous aseptic conditions by a single qualified operator (first author) with ten years of expertise in pediatric endodontics. A digital intraoral photo-simulated phosphorus plate sensor (PSP, Planmeca ProSensor HD, Helsinki, Finland) was used for a perioperative assessment prior to commencing the apexification procedure. A 2% mepivacaine with 1:20,000 levonordefrin (Alexandria Co., Egypt) was used for teeth anesthesia after that isolation was done using a rubber dam (Midwest Dental, Texas, USA). In the beginning, using sterile no. 330 carbide burs in a high-speed contra-angle handpiece with plenty of water cooling and high suction, the pulp chamber was accessed. Then, the working length was measured as one millimetre less than the apex with the conventional intraoral periapical radiographic method using manual endodontic files (Mani Inc., Japan).

After removing the pulp tissue and gently cleaning the root canal with hand files, the root canal was irrigated with 5 ml of 0.5% sodium hypochlorite and afterward with 5 ml of normal saline. Then, sterile paper points (Dentsply Maillefer, OK, USA) were used to dry the root canal. An intracanal medication, calcium hydroxide with iodoform (Well-Pex, Vericom Co., Korea) was inserted into the root canal. After that, reinforced zinc oxide eugenol (IRM Dentsply International, USA) was used to secure the access cavity. After 7–14 days, the patient was summoned back for follow-up appointments. In the case that the tooth was symptomatic, intracanal medication was substituted, the canal was temporarily sealed, and the patient was directed to return for another appointment in 7–14 days. When the tooth stayed asymptomatic following the first visit or upon the second visit of a medication change, the subsequent visit was an apical plug placement. In each group, the intracanal medication was eliminated by manual instrumentation, and then irrigation with normal saline and dryness with sterile paper points were done.

In the MTA group, the material was introduced into the apical 4 mm of root canals by a hand dental plugger size 40/80 (Fanta Dental Plugger, Fanta Co., Shanghai, China). After that, a wet cotton pellet was inserted, and a glass-ionomer-based restoration (Medfill Promedica) was used to seal the access cavity to allow the complete set of MTA. The MTA paste was made by mixing 0.16 g of MTA powder with sterile saline to produce a uniform paste^23^. On the next visit, local anesthesia was administered, and tooth isolation was done, then the material setting was confirmed using a #40 K-file. After that, the remaining part of the canals was obturated with gutta-percha (Dentsply Maillefer, USA), and the coronal part was restored with glass-ionomer and composite restorations (Filtek Z250, 3 M ESPE, St. Paul, MN, USA).

While in the Well-Root PT group, the material was inserted into the orifice of the pulp chamber by a dental dispenser gun (3 M Filtek Co., Germany), then was introduced for about 4 mm of the apical part of the canal by the same method that was performed in the MTA group. After 12 min^24^, Well-Root PT was set, then gutta-percha was used to fill the coronal and middle thirds of the root canal. The coronal restoration was accomplished with glass-ionomer and composite restorations. In both groups, periapical radiographs were done to confirm that the apical plug was positioned correctly and had the proper thickness before proceeding to gutta-percha obturation. Postoperatively, an intraoral periapical radiograph was obtained under the same standardized settings.

### Clinical and radiographic evaluation

Two skilled, blinded pediatric dentists (N.I.A, S.S.E.) carried out clinical and radiographic evaluations at 6 and 12 months, aligning with a core outcome set for endodontics (COS-ENDO), which standardizes the apexification outcomes^3^. The teeth were clinically evaluated for the presence or absence of spontaneous pain, aberrant movement, soreness with percussion, and swelling. The radiographic evaluation was performed at baseline, six, and twelve months for the periapical radiolucency (PAR) area and bone radiodensity. Also, using the periapical index (PAI) system^25^, the periapical condition on the preoperative and follow-up radiographs was scored as follows: (1) normal periapical structures; (2) small bone structure changes; (3) bone structure changes accompanied by considerable loss of minerals; (4) periodontitis with a distinct radiolucent area; and (5) severe periodontitis with aggravating characteristics.

A photo-simulated phosphorus plate sensor (PSP, Planmeca ProSensor HD, Helsinki, Finland) was used to take direct standardized digital radiographs. The parallel periapical technique was obtained by Rinn (XCP, Eign, IL), which was attached to the X-ray tube with its arm fastened to the film holder that contained a coated PSP (photo-stimulated phosphorus plate). To ensure standardization during radiographic film retakes, condensation rubber base impression material was positioned on the outside of the film holder, and the child was told to bite on it while the material was set. The sensor was exposed to an X-ray machine (Planmeca ProX, Helsinki, Finland) with a central ray perpendicular to the sensor at 70 kVp, 6 mA, and 0.8 s. Radio-densitometric and radiometric analysis of the radiographs was performed by RadiAnt software (RadiAnt DICOM Viewer 2025.1, Medixant, Poland, https://www.radiantviewer.com). Radiometric analysis using the closed polygon tool to measure the radiolucent area related to the periapical region, which was less dense than normal bone before treatment, 6-, and 12-month post-treatment in both groups. While radio-densitometric analysis of bone density was evaluated by selecting the regions of interest (ROIs) just periapical and centralized to the root apex of the affected tooth to measure the mean value of gray level using arrow (which measures density scale between 0 and 256 with being zero lowest value and 256 highest dense value) of the pixel related to ROIs before treatment, 6-, and 12-months post-treatment in both groups. It should be kept in consideration that the gray value of the pixel of ROIs was just apical and centralized to the apex of the affected tooth, and its primary calibration of this tool depends on the KVp, which was always kept constant^26^.

### Statistical analysis

The statistical package for social studies (SPSS version 26, developed by IBM, Illinois, Chicago, USA) was used to organize, tabulate, and statistically analyze the gathered data. For numerical variables, the mean, standard deviations, and range were computed. The student’s t-test was utilized to determine the differences between the two mean values. Repeated measurements analysis of variance was done to test differences in observations during the follow-up period, and when found significant, a Bonferroni test was performed for pairwise analysis. For categorical variables, the number and percentage were computed, and the chi-squared test was used to compare subcategories. When the chi-squared test was not applicable due to small observations leading to having more than 20% of cells with expected values < 5, the Monte Carlo exact test was used. A comparison of periapical index scores at different follow-up periods within each group was done using the Friedman test. The significance level was set at *p* < 0.05.

## Results

Table 1 Displayed all demographic information, including the age and gender distributions of the children recruited in each group. Kappa scores for all assessments were greater than 0.87, indicating a high level of inter-examiner consistency. All patients were presented at 6- and 12-month follow-ups for clinical and radiographic evaluations. Group-I included 48% females and 52% males with a mean age of 8.79 *±* 0.65 years, while group-II included 52% females and 48% males with a mean age of 8.61 *±* 0.58 years, with no significant statistical difference between groups.

**Table 1:** Demographic distribution of study participants.

Variables	MTA		Well-Root PT		Test	*p*
	*n*	%	*n*	%		
Age in years					t = 1.033	0.307
Range	8.0–10.0		8.0-9.5			
Mean *±* SD	8.79 *±* 0.65		8.61 *±* 0.58			
Sex					*X*^*2*^ = 0.080	0.777
Males	13	52.0	12	48.0		
Females	12	48.0	13	52.0		

### Clinical evaluation

Both groups showed improved clinical signs and symptoms throughout all follow-up periods, with non-significant statistical differences between the two groups. In the Well-Root PT group, at six months, one case (4%) sensed spontaneous pain, tenderness on percussion, and mobility, while at twelve months, all cases had no signs or symptoms of irreversible pulpitis. Regarding the MTA group, at six months, four cases (16%) had spontaneous pain and tenderness on percussion, with only one case still having the same symptoms at twelve months follow-up. As revealed in Table 2, the Friedman test revealed significant statistical differences for all clinical variables within the same group at various follow-up periods (*P* < 0.05).

**Table 2:** Clinical findings in both groups at various follow-up periods.

Clinical criteria	MTA (*n* = 25)		Well-Root PT (*n* = 25)		*p*
	*n*	%	*n*	%	
Spontaneous pain:					
Baseline	20	80.0	21	84.0	1.000
At 6 months	4	16.0	1	4.0	0.349
At 12 months	1	4.0	0	0.0	1.000
*Friedman X*^*2*^	32.947		40.095		
*p*	< 0.001*		< 0.001*		
Tenderness on percussion:					
Baseline	20	80.0	21	84.0	1.000
At 6 months	4	16.0	1	4.0	0.349
At 12 months	1	4.0	0	0.0	1.000
*Friedman X*^*2*^	32.947		40.095		
*p*	< 0.001*		< 0.001*		
Mobility:					
Baseline	6	24.0	5	20.0	1.000
At 6 months	2	8.0	1	4.0	1.000
At 12 months	1	4.0	0	0.0	1.000
*Friedman X*^*2*^	8.400		8.400		
*p*	0.015*		0.015*		
Swelling:					
Baseline	5	20.0	4	16.0	1.000
At 6 months	0	0.0	0	0.0	1.000
At 12 months	0	0.0	0	0.0	1.000
*Friedman X*^*2*^	10.000		8.000		
*p*	0.007*		0.018*		
Abscess/fistulation					
Baseline	5	20.0	4	16.0	1.000
At 6 months	0	0.0	0	0.0	1.000
At 12 months	0	0.0	0	0.0	1.000
*Friedman X*^*2*^	10.000		8.000		
*p*	0.007*		0.018*		

*significant

### Radiographic evaluation

**Fig. 2:**
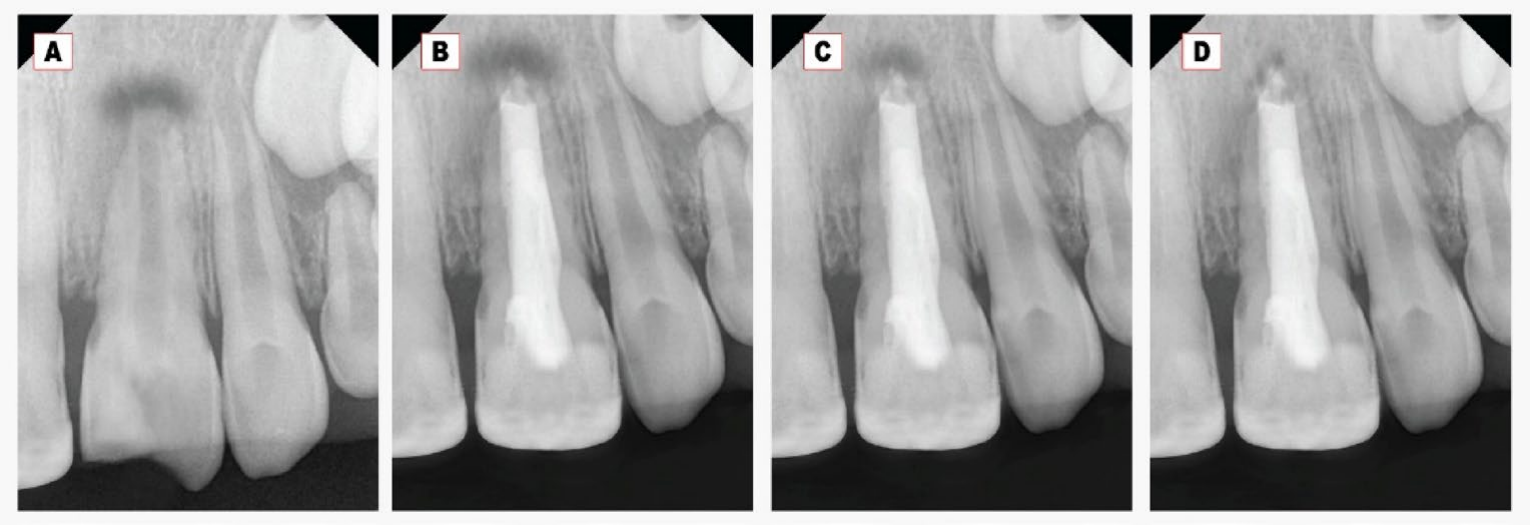
**A**: Preoperative intraoral peri-apical (IOPA) radiograph of upper left permanent central incisor. **B**: Immediate IOPA radiograph after MTA plug and gutta-percha obturation. **C-D**; Post-IOPA radiographs at 6 and 12 months.

**Fig. 3:**
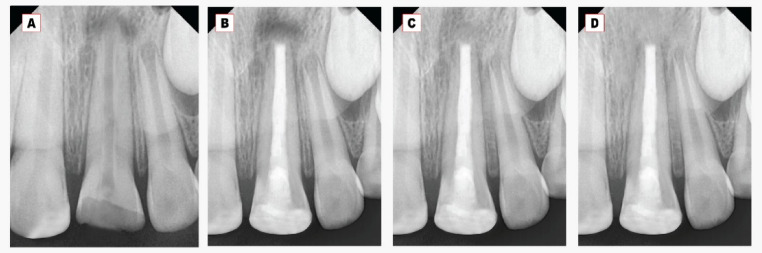
**A**: Preoperative intraoral peri-apical (IOPA) radiograph of upper left permanent central incisor. **B**: Immediate IOPA radiograph after Well-Root PT plug and gutta-percha obturation. **C-D**; Post-IOPA radiographs at 6 and 12 months.

Regarding the periapical radiolucency (PAR) area, a significant decrease in the PAR area was found at all follow-ups within each group Figs. 2 and 3. At twelve months, the mean PAR area in the Well-Root PT group was (0.14 *±* 0.08) compared to (2.3 *±* 0.9) in the MTA group. A highly statistically significant difference was observed between the two tested groups at all follow-up periods (*p* < 0.001) as represented in Table 3.

**Table 3:** Comparison of periapical radio-translucency area and bone radiodensity between both groups at different follow-up periods.

Radiographic Variables	MTA (*n* = 25)		Well-Root PT (*n* = 25)		t	*p*
	Range	Mean *±* SD	Range	Mean *±* SD		
Periapical radiolucency area						
Baseline	17–23	20.34 *±* 1.22	17–23	20.9 *±* 1.1	1.748	0.087
At 6 months	10–16.	13.1 *±* 1.6	8–15	10.9 *±* 1.5	4.959	< 0.001*
At 12 months	0-4.4	2.3 *±* 0.9	0-0.3	0.14 *±* 0.08	11.168	< 0.001*
*F#*	1926		4102.0			
*p*	< 0.001*		< 0.001*			
Periapical bone radiodensity						
Baseline	109–133	118.4 *±* 6.3	108–128	118.6 *±* 5.6	0.142	0.887
At 6 months	122–143	133.1 *±* 5.8	122–155	142.8 *±* 7.1	5.277	< 0.001*
At 12 months	159–174	164.8 *±* 9.4	166–186	178.2 *±* 5.4	10.604	< 0.001*
*F#*	839.1		1352.8			
*p*	< 0.001*		< 0.001*			

*Significant.

^#^Bonferroni test: each observation was significantly different from the other two observations.

Concerning the periapical bone radiodensity, there was a significant increase in the bone radiodensity at 6- and 12-month follow-up periods within each group, as shown in Table 3 and Figs. 2, 3. The mean periapical bone radiodensity in the Well-Root PT group was (178.2 *±* 5.4) compared to (164.8 *±* 9.4) in the MTA group at twelve-month follow-up. A highly statistically significant difference was found between the MTA and Well-Root PT groups at all follow-up periods (*p* < 0.001). Using the Bonferroni test, each observation was significantly different from the baseline and the other observation (*p* < 0.001).

Interpreting the periapical index (PAI) scores, all teeth in both groups exhibited progressive healing of the periapical lesions, with no statistically significant differences noted between the two groups during 6 and 12-follow-up intervals Table 4. Using the Friedmann test, there was a highly statistically significant improvement in the healing of periapical lesions within each group (*p* < 0.001).

**Table 4:** Comparison of the periapical index scores between both groups at different follow-up periods.

Periapical index scores	MTA (*n* = 25)	Well-Root PT (*n* = 25)	*p*-value#
Baseline:			**1.000**
1	0	0	
2	0	0	
3	3	2	
4	21	22	
5	1	1	
At 6 months			**0.129**
1	2	5	
2	16	18	
3	7	2	
4	0	0	
5	0	0	
At 12 months			**0.289**
1	18	22	
2	7	3	
3	0	0	
4	0	0	
5	0	0	
*Friedman test*	**47.895**	**47.574**	
*p*	< 0.001*	< 0.001*	

*Significant, ^#^Monte Carlo exact test

## Discussion

Treating immature permanent teeth with an open apex presents challenges, including thin walls that are prone to fracture and the absence of an apical stop, which increases the risk of material extrusion during treatment. Therefore, apexification must be performed cautiously using appropriate materials^27^. Ensuring the apical sealing of bioactive endodontic material used as an apical plug is crucial for the clinical success of apexification, as it prevents microorganisms from reinfecting the root canal system^28^. While MTA provides effective sealing against microbial leakage, it has drawbacks such as a prolonged setting time, the risk of dentin discoloration, and challenges in handling^29^. Its extended setting time may increase the likelihood of bacterial leakage^30^. Additionally, the apical seal may be compromised by voids caused by air bubbles, pores, or capillary channels present in the hardened cement^31^. To overcome MTA limitations, the innovative premixed material Well-Root PT offers exceptional biocompatibility, strong sealing capability, and effective antibacterial properties^17^. Unlike MTA, its premixed formulation provides benefits such as improved uniformity and the convenience of injecting only the needed amount, minimizing material waste^32^. Additionally, its quick setting time is particularly advantageous in pediatric treatments, as it helps reduce the procedure duration and the potential for disruptive behavior^33^. So, this study was directed to evaluate and compare the clinical and radiographic outcomes of using Well-Root PT as an apical plug versus mineral trioxide aggregate (MTA) in the treatment of nonvital immature permanent incisors.

The clinical and radiographic evaluations in the present study were aligned with a core outcome set for endodontics (COS-ENDO) created by Zanjir et al.^3^, which standardizes the apexification outcomes confirming consistent reporting and improving research efficiency.

The current study findings accepted the null hypothesis regarding the clinical success of the two tested groups, as no statistically significant differences were observed across all follow-up periods. However, a significant difference was noted in the periapical radiolucency area and periapical bone radiodensity between both groups at all follow-up intervals (The null hypothesis was partly refuted).

The present study results revealed that both groups showed improved clinical signs and symptoms during all follow-up periods with non-significant statistical difference; This can be attributed to the alkaline pH of these materials and their ability to release Ca²⁺ ions, which can eliminate bacteria and promote the remineralization process^34^. This agreed with Barakat and Fathi^35^., who found that the success rates for the bioceramic root canal sealer and MTA were 93.3% and 90%, respectively, with non-significant statistical differences. Also, this coincided with Shaker et al.^36^, who found after 12 months, no patients in either of the two tested groups (Well-Root PT, MTA Biorep) experienced pain when biting or during percussion, nor did they exhibit swelling, abscesses, sinus tracts, or abnormal tooth mobility. Furthermore, the present study results were in line with Anjum et al.^37^, who revealed that Biodentine had a similar success rate as MTA (85.0% vs. 90.9%) when used as apical plugs in non-vital immature permanent incisors. Moreover, this matched the results of the Yadav et al.^1^, study in which the clinical success of MTA, Biodentine, and calcium phosphate cement in a single-visit apexification procedure was 100%. Another study conducted by Santos et al.^4^, also, agreed with the current study’s findings, which evaluated the long-term outcome of apexification procedures in 16 nonvital immature permanent maxillary central incisors that were treated with MTA, Biodentine, or β-tricalcium phosphate within a follow-up duration of 5 to 22 years and it was concluded that only one tooth was lost due to root resorption, and one patient exhibited with clinical signs and symptoms upon recall, giving in a 93.8% survival rate.

In the Well-Root PT group, at twelve months, all cases revealed no signs or symptoms of irreversible pulpitis; this concurred with Chae et al.^19^, who stated that Well-Root PT possesses antibacterial properties by creating an alkaline environment post-application. Also, this was consistent with Back et al.^33^, who found that Well-Root PT demonstrated significantly higher pH values compared to TheraCal LC and Ultra-Blend Plus (a resin-modified calcium hydroxide cement) across all measurement periods (*p* < 0.05) additionally, its pH increased significantly over time after setting, stabilizing after one day. On the other hand, in the MTA group in this study, one case still had the same symptoms at twelve months of follow-up; this could be attributed to the persistence of the original infection, likely due to the wide pulp space and challenges in effective debridement as a result, residual bacteria, and tissue breakdown products may remain, hindering the healing process^37^.

Regarding the periapical radiolucency (PAR) area, there were highly statistically significant differences between the two tested groups at all follow-up periods (*p* < 0.001), with the highest decrease reported in the Well-Root PT group. This may be clarified through the fact that Well-Root PT is a premixed material with a consistent composition, offering the benefit of highly reliable treatment outcomes^18^. Also, the physical and chemical properties of MTA may differ depending on the powder/liquid ratio; these alterations in physical and chemical properties may lead to extended setting time, increased solubility, reduced mechanical hardness, and decreased calcium ion release^38^. This was in line with Barakat and Fathi^35^., who reported that reduced radiolucency and clear evidence of bone healing were observed in 93.3% of teeth treated with a bioceramic root canal sealer and 90% of teeth in the MTA group. Additionally, this coincided with Shaker et al.^36^, who reported after one year of follow-up, the lesion size in the MTA group had decreased by 91%, while in the Well-Root PT group, it was 93%, with no statistically significant differences. On the other hand, this conflicted with Singla et al.^39^, who found that the resolution rate of radiolucency was similar among the Biodentine, MTA, and calcium hydroxide groups in terms of periradicular healing and root-end calcific tissue repair in traumatized immature permanent anterior teeth, with non-significant statistical differences between the groups.

Additionally, there was a significant decrease in the periapical radiolucency (PAR) area within the MTA group at all follow-ups; this agreed with Pace et al.^40^, who found that the periapical index (PAI) score decreased significantly between baseline and one-year follow-up also between one and five years. This could be attributed to MTA’s excellent sealing properties, biocompatibility, and dimensional stability. Additionally, MTA achieves a pH of 12.5 after setting, comparable to calcium hydroxide, which is believed to confer antimicrobial properties^41,42^. Moreover, the present study results revealed that all teeth in both groups exhibited progressive healing of the periapical lesions, with no statistically significant differences between the two groups; this was consistent with Tolibah et al.^43^, who found no statistically significant differences in the periapical index (PAI) scores between the MTA and Biodentine groups in treating nonvital immature first permanent molars at 6 and 12 months follow-up. Also, this was in agreement with Santos et al.^4^, findings that ten cases of 16 nonvital immature permanent maxillary central incisors were deemed radiographic successes based on the periapical index (PAI) system (five cases were evaluated as PAI 1 and five as PAI 2), and six cases had apical periodontitis (three cases scored as PAI 3, two cases as PAI 4, and one case as PAI 5) within a follow-up duration of 5 to 22 years.

Concerning the periapical bone radiodensity, within each group, the bone radiodensity increased significantly during the 6- and 12-month follow-up periods; this may be attributed to the amount and balance of osteoblasts and osteoclasts which determine how the bone tissue around injured teeth heals^44^. MTA strongly reduces RANKL-mediated osteoclastogenesis and osteoclast activity, reducing bone resorption in periapical lesions^45^.

Furthermore, bioceramics affect the proliferation, differentiation, migration, and death of stem cells, osteoblasts/osteoclasts, dental pulp cells (DPCs)/periodontal ligament cells (PDLCs), and immune cells^46^. Also, the interaction between bioceramic materials and cells is crucial for controlling inflammation and promoting wound repair^47^.

Moreover, this study’s findings revealed that the mean periapical bone radiodensity in the Well-Root PT group was (178.2 *±* 5.4) compared to (164.8 *±* 9.4) in the MTA group at twelve-month follow-up, with highly statistically significant differences (*p* < 0.001). This could be due to the comparable bioactive potential of Well-Root PT to MTA in hard tissue formation, also increased calcium ion release enhances the osteoblast differentiation, leading to a higher pH that supports the formation of hard tissue^48^. This was consistent with an immunohistochemical study conducted by Chae et al.^19^, who used the pulp inflammation (CD68) and hard tissue formation (DSPP) markers to assess the biocompatibility and bioactivity of Well-Root PT; they found that CD68-positive area was 2.4 *±* 0.6% in MTA, 2.5 *±* 2.0% in Biodentine, and 3.4 *±* 2.1% in Well-Root PT on day 7. On day 28, the DSPP-positive area was 5.2 *±* 3.0% in MTA, 5.2 *±* 1.9% in Biodentine, and 6.1 *±*1.9% in Well-Root PT. Also, this was in line with Sun et al.^49^, who assessed the effect of bioceramic iRoot putty (iRoot FS) on the proliferation, migration, and differentiation of human dental pulp stem cells (hDPSCs) and revealed that it enhanced the proliferation, migration, and osteogenic differentiation of hDPSCs compared with Biodentine. Moreover, Lv et al.^50^, evaluated the biocompatibility of innovative bioceramic (iRoot FS) and compared its performance to MTA and found that bioceramic (iRoot FS) induced higher viability of MC3T3-E1 osteoblast cells compared to MTA.

The limitations of this study include firstly, the sample size, although statistically justified, was relatively small also, a relatively short follow-up period of 12 months, which may not be sufficient to evaluate the complete range of outcomes. Moreover, periapical radiographs used for evaluation, though standard, have limitations in detecting three-dimensional healing and may not fully represent periapical tissue response compared to more advanced imaging like Cone Beam Computed Tomography (CBCT), which may not have been used routinely due to radiation concerns and high cost. Further clinical research with larger sample sizes and longer follow-up periods is required to assess the risks, benefits, and success of these materials and validate the findings of this study.

The findings of this study have significant clinical implications in the management of nonvital immature permanent teeth. The comparable clinical and radiographic success rates of premixed bioceramic putty (Well-Root PT) and MTA suggest that both materials are effective options for achieving apical closure. However, the use of premixed bioceramic putty (Well-Root PT) offers practical advantages in clinical settings, including simplified handling, reduced procedure time, and elimination of mixing errors, which can enhance workflow efficiency and consistency in outcomes. These benefits may be particularly valuable in pediatric or anxious patients, where minimizing chair time is crucial. Additionally, the favorable sealing ability and biocompatibility of premixed bioceramic materials support their use as a reliable alternative to MTA in cases requiring apical plug placement.

## Conclusion

Within the limitations of this study, it was concluded that:

- Both groups showed improved clinical signs and symptoms during all follow-up periods with no statistically significant difference.
- Well-Root PT showed a significant decrease in the periapical radiolucency area at all follow-ups compared to MTA.
- Well-Root PT reported a significant increase in the periapical bone radiodensity at all follow-ups.
- Well-Root PT demonstrates satisfactory clinical and radiographic success as an apical plug for nonvital immature permanent incisors, providing enhanced operability and reduced technical sensitivity.

## Data Availability

The datasets used and/or analysed in this study are available from the corresponding author upon reasonable request.
